# Low Serum Vitamin E Level Associated with Low Hand Grip Strength in Community-Dwelling Adults: Korean National Health and Nutrition Examination Survey (KNHANES VII) 2016–2018

**DOI:** 10.3390/nu13051598

**Published:** 2021-05-11

**Authors:** Yongjae Kim, Sungjae Shin, Namki Hong, Yumie Rhee

**Affiliations:** 1 Division of Medicine, Yonsei University College of Medicine, Seoul 03722, Korea; smart1777@naver.com; 2 Division of Endocrinology, Department of Internal Medicine, Endocrine Research Institute, Yonsei University College of Medicine, Seoul 03722, Korea; ssj6310@naver.com (S.S.); YUMIE@yuhs.ac (Y.R.)

**Keywords:** antioxidant, grip strength, sarcopenia, tocopherol, vitamin E

## Abstract

This study assessed the association between serum vitamin E levels and hand grip strength (HGS) in community-dwelling adults data of 1011 men aged 50 years and older and 1144 postmenopausal women were analyzed. Low HGS was defined as HGS below the sex-stratified median value. Proportion of low HGS was the greatest in the lowest quintile of serum vitamin E level (<10.51 mg/L, 57.1%), with a decreasing trend toward the highest vitamin E quintile (>17.81 mg/L, 43.6%; *p* < 0.001). A one-unit (mg/L) decrease in vitamin E levels was associated with lower HGS in men (adjusted beta coefficient −0.10, 95% confidence interval [CI] −0.18 to −0.02, *p* = 0.019), but not in women (−0.01, 95% CI −0.06 to 0.03, *p* = 0.550). Compared with the middle quintile (Q3; 12.59–14.69 mg/L), the lowest vitamin E quintile (Q1) was associated with elevated odds of low HGS (adjusted odds ratio [aOR]: 1.38, *p* = 0.045), independent of sociodemographic factors, health-related lifestyles, comorbidities, dietary intake, and cholesterol level. However, the odds of low HGS did not differ significantly in other vitamin E quintiles (Q2, aOR 1.12; Q4, aOR 1.38; Q5, aOR 1.12; *p* > 0.05). Individuals with the lowest quintile vitamin E level had elevated odds of low HGS independent of covariates, findings which merit further validation.

## 1. Introduction

Hand grip strength (HGS) is a basic tool for evaluating the physical performance of older adults. Due to of its convenience, it is widely used in medical settings [1,2]. Previous studies have shown that HGS is associated with indicators of nutritional status. In addition, low HGS is associated with increased mortality, cognitive impairment, and anemia [3,4,5,6]. HGS is used as a diagnostic tool for sarcopenia and frailty syndrome, which are typical age-related diseases [7,8,9].

A recent paper reported that the amount of vitamin E (α -tocopherol/d) consumed by Korean men was 6.5 mg/d and that consumed by Korean women was 5.7 mg/d, which is 62% and 53% of the adequate intake according to Dietary Reference Intakes for Koreans 2015 [10]. In another study of 20 to 59-year-old adults living in the Seoul metropolitan area of South Korea, 23% were vitamin E deficient with serum α -tocopherol <12 μmol/L (mean serum α -tocopherol in men: 15.4, women: 15.0) [11,12]. Sarcopenia is a representative aging-related disease characterized by progressive and generalized loss of skeletal muscle mass and strength. Its pathophysiology is known as oxidative stress, chronic inflammation, and mitochondrial dysfunction, and as vitamin E is known as antioxidants, vitamin E deficiency can aggravate the disease [13]. In addition, low vitamin E status might be associated with the risk of coronary heart disease, eye disorders (age-related macular degeneration and cataracts), cognitive decline, and certain types of cancers [14,15,16,17].

A few papers have researched the association between serum vitamin E level and age-related diseases, such as sarcopenia and frailty syndrome. In a paper that studied the correlation between serum vitamin E and indicators of sarcopenia, lower vitamin E level was independently associated with lower muscle strength [18]. Likewise, in another paper, low vitamin E level was associated with an increased risk of frailty syndrome [19]. In contrast, a recent paper investigating the association between biochemical nutrient status markers and muscle parameters stated there was no significant association between serum vitamin E level and HGS [20]. However, these studies were limited by small sample size or nonrepresentative samples.

Thus, our study aimed to assess the cross-sectional association between serum vitamin E level and HGS in healthy community-dwelling adults. We used representative, reliable data for all Koreans, which included objective measures of both nutritional status and physical function.

## 2. Materials and Methods

### 2.1. Study Population

**Figure 1:**
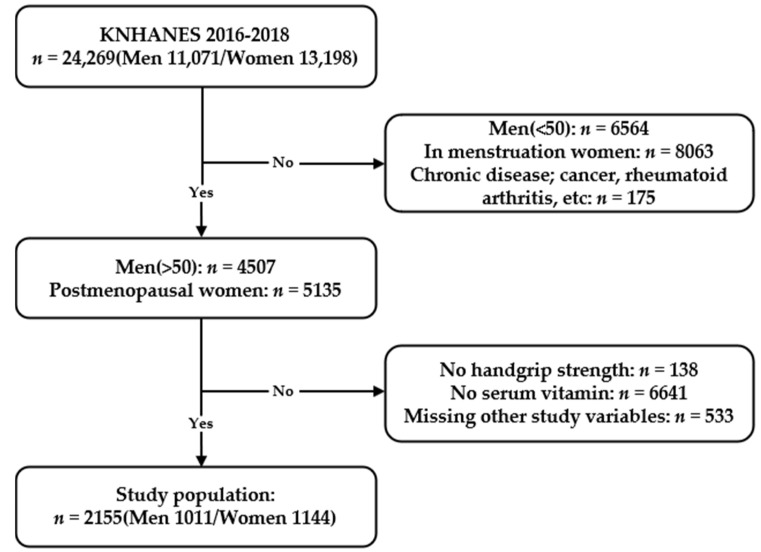
Flowchart of the study population from the Korean National Health and Nutrition Examination Survey VII (2016–2018) showing inclusions and exclusions.

The Korea National Health and Nutritional Examination Survey (KNHANES) is a nation-wide, cross-sectional study with national representativeness. The KNHANES contains data including participants’ overall lifestyle, anthropometric measurements, blood tests, answers from health-related questionnaires, and nutritional surveys. All survey protocols were approved by the institutional review board of the Korea Centers for Disease Control and Prevention (approval numbers: 2018-01-03-P-A):

A total of 24,269 participants were enrolled in KNHANES VII (2016–2018). Of these participants, 11,071 were men and 13,198 were women. Since sarcopenia is known to start mainly in people in their 50s [7,21], we selected from this population men aged ≥50 years (*n* = 4507) and postmenopausal women (*n* = 5135). A self-reported questionnaire was used to select the postmenopausal women. People without data for serum vitamin E or HGS were excluded, leaving 2863 participants. People without additional data needed for statistical analyses were further excluded, leaving 2330 participants. These data include height, weight, smoking habits, drinking habits, serum high-sensitivity C-reactive protein (hsCRP) levels, fasting serum glucose levels, total cholesterol levels, educational status, household income, frequency of resistance exercise, use of dietary supplements, and comorbidities such as diabetes, hypertension, and anemia. People with systemic diseases such as various cancers, rheumatoid arthritis, stroke, liver cirrhosis, and kidney failure were excluded from the study population leaving 2155 people. Finally, 2155 people participated in the study; 1011 were men and 1144 were women (Figure 1).

### 2.2. Hand Grip Strength

HGS was measured by a Takei digital grip strength dynamometer (T.K.K. 5401, Takei Scientific Instruments Co. Ltd., Tokyo, Japan). People without arms, hands, or fingers, people with broken fingers, people with paralyzed hands, and people with casts or bandages for their wrists or hands were excluded from measuring HGS. Participants were told to stand up and look straight ahead with both arms falling naturally. Elbows and wrists were not to be flexed, and arms not to be in contact with the participants’ bodies. The feet were to be hip-width apart and evenly spaced. The first measured HGS was for the preferred hand of the participant. In turn, the HGS of the other hand was measured. This was repeated three times to get three HGS values for each hand. After one measuring cycle, participants took a 60-s break before the next cycle of measurements. Before each measurement, participants were checked for correct posture. Each measurement took place after inhaling and the maximum HGS was measured for 3 s while exhaling. We defined HGS as the maximum value from six measured values from both hands [2]. We defined people with low HGS as having lower HGS than the sex-specific median values, <38.3 kg for men and <22.8 kg for women, respectively.

### 2.3. Study Variables

Height and weight were obtained while participants wore disposable non-woven clothing without shoes or socks. Body mass index (BMI) was calculated from height and weight. Household income, current smoking status, educational status, frequency of resistance exercise, and frequency of alcohol consumption were assessed by self-reported questionnaires and categorized into study variables. Household income was categorized into two levels by age group-specific medians. Regular alcohol consumption was defined as drinking alcohol once or more a month. Educational status was divided into <high school and ≥high school. The frequency of resistance exercise was categorized into three levels. None, intermittent, and regular resistance exercises were defined as exercising 0 times a week, 1–3 times a week, and ≥4 times a week, respectively. The 24-h dietary recalls were conducted through in-person interviews by trained dietary staff in mobile examination centers to explore food kinds and amounts that the subjects consumed for the past 24 h (midnight to midnight). Daily intakes of calories and nutrients were calculated from the food intake information acquired from the interviews using the Can-Pro 2.0 nutrient intake assessment software developed by the Korean Nutrition Society [22]. Dietary supplement usage was defined as “Yes” if the participant consistently used dietary supplements of any kind for 2 weeks in the past year. Diabetes was defined as having a fasting serum glucose level >126 mg/dL, doctor diagnosis, or on diabetes medication [23]. Hypertension was defined as having systolic blood pressure >140 mmHg, diastolic blood pressure >90 mmHg, or on hypertension medication [24]. Anemia was defined as having a hemoglobin level <12 g/dL for women, and <13 g/dL for men [25].

After 8 h of fasting, blood was drawn from the median cubital vein or cephalic vein for various biomedical analyses. Hemophiliacs, people on anticoagulants, people who had received chemotherapy within a month, people aged ≥80 years, and people with shunts for hemodialysis were excluded from drawing blood. People with rashes on both arms, open wounds, paralysis, and vascular problems were also excluded. Fasting serum glucose level was measured by the hexokinase UV method, and serum total cholesterol level was measured by the enzymatic method. Both markers were measured using the Hitachi Automatic Analyzer 7600-210 (Hitachi, Tokyo, Japan). Serum hsCRP level was measured by immunoturbidimetry using Cobas (Roche, Mannheim, Germany). Serum vitamin E level was measured by a high-performance liquid chromatography-flame ionization detector using Agilent 1200 (Agilent Technologies Inc., Santa Clara, CA, USA). The reference normal range for serum vitamin E level in adults is 5.0–20.0 mg/L. The coefficient of variation of control material was <9% for low level (5.0 mg/L) and <14% for high level (20.0 mg/L), respectively. All values are lower than 15%, the allowable range set by Korea Disease Control and Prevention Agency [26]. The health interview and health examination are performed by trained medical staff and interviewers at the mobile examination center [27].

### 2.4. Statistical Analyses

The baseline characteristics of the study population were assessed according to serum vitamin E quintiles. This analysis was performed using the one-way analysis of variance (ANOVA) for continuous variables and χ² (chi-square) test for categorical variables. A post-hoc analysis for ANOVA was performed by Bonferroni correction. In addition, the Cochran-Armitage trend test was performed to assess the serial trend of low HGS according to the vitamin E quintiles. For hsCRP, an analysis was performed by the Kruskal–Wallis test and the post-hoc analysis for this test was performed with the Dunn procedure. Based on clinical backgrounds and previous studies, we selected potential determinants for low HGS. Using these determinants, multivariable linear regression models and multivariable logistic regression models were constructed to examine the relationships between serum vitamin E levels and HGS. Four models progressively containing more adjusting variables were constructed: Model 1 adjusted for basic factors such as age, sex, and BMI; Model 2 further included socioeconomic factors related to health such as household income, alcohol consumption, current smoking status, and frequency of resistance exercise; Model 3 additionally included comorbidities (hypertension and diabetes) and laboratory values (serum total cholesterol and log-transformed hsCRP; serum hsCRP was log-transformed due to its skewed distribution); and Model 4 further adjusted for factors reflecting a participant’s nutritional status such as total calorie intake and use of dietary supplements. A local polynomial regression model was used to detect potential nonlinearity in the association between serum vitamin E level and the probability of low HGS. Statistical analyses were performed using STATA 14.1 (Stata Corp., College Station, TX, USA) [28]. A *p*-value of <0.05 was considered statistically significant.

## 3. Results

### 3.1. Study Subjects

The baseline characteristics of the study population are presented in Table 1. The mean age (±standard deviation) and BMI of the total participants were 63.1 ± 8.9 years and 24.1 ± 3.0 kg/m^2^, respectively. The mean HGSs were 37.7 ± 7.3 kg for men and 22.7 ± 5.0 kg for women. Participants were grouped into quintiles for serum vitamin E levels. Proportion of low HGS was the greatest in the lowest quintile (Q1, 57.1%) and showed a steadily decreasing trend through the quintiles reaching the highest quintile (Q5, 43.6%; *p*-for-trend < 0.001). For continuous variables, Q1 was significantly old (64.5 ± 9.3 vs. 62.0 ± 8.4 years, post-hoc *p* < 0.001), much taller (162.2 ± 8.3 cm vs. 160.1 ± 8.5 cm, post-hoc *p*-value < 0.001), and have low cholesterol level (164.4 ± 33.5 vs. 219.6 ± 42.2 mg/dL, post-hoc *p*-value < 0.001) compared to Q5. Particularly in the lowest quintle (Q1), the percentage of women, people with high household income, and dietary supplement usage, current smokers, anemia, and diabetes were significantly greater compared with other quintiles.

### 3.2. Association between Serum Vitamin E and Low Hand Grip Strength

In a multivariable linear regression model, a 1-unit (mg/L) decrease in serum vitamin E level was associated with lower HGS in men (adjusted beta coefficient −0.10, 95% confidence interval [CI] −0.18 to −0.02, *p* = 0.019), whereas the association was not significant in women (−0.01, 95% CI −0.06 to 0.03, *p* = 0.550).

**Figure 2:**
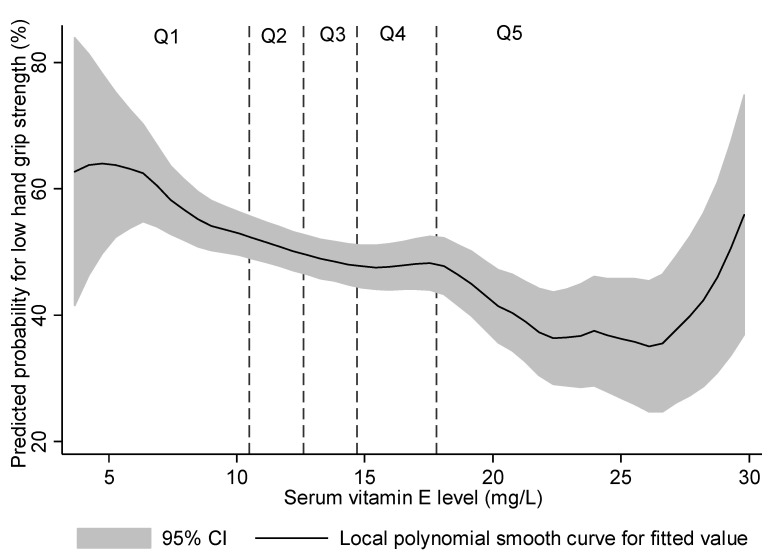
Predicted probability for low hand grip strength (below the sex-stratified median) using a local polynomial smooth curve.

A non-linear probability for low HGS was fitted against serum vitamin E level in Figure 2. The probability of low HGS was elevated in the lowest quintile range (Q1, <10.51 mg/L), whereas the probability of low HGS remained stable across the mid-quintiles (Q2 to Q4), with a U-shaped association at the highest quintile (Q5).

### 3.3. Association between Serum Vitamin E Quintiles and Hand Grip Strength

Multivariable logistic regression analyses were conducted to reveal the independent association between serum vitamin E quintiles and low HGS, with the middle quintile (Q3) as the referent group (Table 2). From the analysis of the final model (Model 4), the lowest quintile (Q1) was associated with elevated odds of low HGS compared with the middle quintile (Q3) (adjusted odds ratio: 1.38, (95% CI: 1.01–1.89), *p* = 0.045) independent of age, sex, BMI, household income, alcohol consumption, current smoking status, frequency of resistance exercises, hypertension, diabetes, serum total cholesterol, log-transformed hsCRP, total calorie intake, and dietary supplement usage. The odds of low HGS did not differ in the other quintiles (Q2, Q4, and Q5) compared with the middle quintile (Q3).

## 4. Discussion

In our study, we assessed the association between quintiles of serum vitamin E level and HGS among a large representative population of Korean community-dwelling adults. The key finding was that the lowest quintile (Q1) was associated with greater odds of low.

HGS compared with the middle quintile (Q3) after adjusting for sociodemographic factors, health-related lifestyles, comorbidities, dietary intake, and lipid profile.

The positive association in our study is supported by several previous human studies investigating the correlation of serum vitamin E level with physical performance and skeletal muscle strength. In the Women’s Health and Aging Studies (WHAS), the participants were severely disabled community-dwelling women aged 70–79 years, and the lowest quartile of serum α-tocopherol was associated with a greater risk of low HGS [19]. In the InCHIANTI study, serum α -tocopherol was significantly correlated with knee extension strength and the physical performance tests (walking speed, ability to stand from a chair, and balancing) in adults aged ≥65 years [29]. Another study conducted with the same InCHIANTI group further observed an association between low circulating serum vitamin E level and frailty syndrome [19]. However, in MaSS, a recent study of community-dwelling adults (aged ≥65 years), no association between quartiles of α-tocopherol/cholesterol ratio and HGS was found [20]. Our study differed in that we compared the association between serum vitamin E and HGS in medium aged community-dwelling adults >50 years old, known as the age at which sarcopenia begins [7,21].

Additionally, several recent studies investigated the association between the intake of vitamin E supplement and HGS. Welch et al. [30] researched the association between dietary antioxidant vitamins and sarcopenic indices in women aged 18–79 years, and vitamin E supplementation was positively associated with fat-free mass, but not with HGS [30]. A study of community-dwelling Swiss adults conducted in the CoLaus study failed to demonstrate the association between self-reported vitamin C+E supplementation and HGS [31]. The Hertfordshire Cohort Study, studied diet and its relationship with HGS in men and women aged 59–73 years and found no association between vitamin E intake and HGS [32]. Those studies did not measure the serum vitamin E level and are not able to confirm whether it was insufficient. Also, vitamin intake may not be correlated with serum concentrations due to genetic variations in vitamin E metabolism [33].

Up-regulated oxidative stress and inflammation play a critical role in age-associated skeletal muscle dysfunction [34,35]. In animal studies, vitamin E has been shown to improve antioxidant defense, suppress oxidative stress, and improve the insulin sensitivity of skeletal muscles [36,37,38]. In human studies, Meydani et al. [39] found out that vitamin E supplementation reduced the expression of oxidative stress markers in both younger (aged 22–29 years) and older (aged 55–74 years) sedentary men. However, most of the papers related to vitamin E supplementation have not proven their effectiveness and have not been valued in clinical practice [30,31,32]. From our results, the lowest quintile (Q1) was associated with elevated odds of low HGS compared with the middle quintile (Q3). On the other hand, if the serum vitamin E level increased beyond the middle quintile (Q3), the local polynomial regression model showed a nonlinear association with low HGS. It suggests the possibility of a threshold effect. There are existing studies that report that vitamins such as E, D, and B12 might have a threshold effect [40,41,42]. Our research shows that vitamin E supplementation may be beneficial for sarcopenia in patients with a lower vitamin E level. Yet, it is difficult to provide evidence that supplementation of vitamin E to a patient whose vitamin E level is within the appropriate range would be helpful. Perhaps these findings may explain some of the controversies in the above studies.

To the best of our knowledge, this is the first study to have evaluated the effect of low concentration of vitamin E on HGS in an Asian population. Our study is based on the nationally representative, community-derived dataset of KNHANES. One strength of our study is its generalizability because the study subjects were healthy community-dwelling adults and could represent the general population. Only a small proportion of people had low HGS based on the HGS cut-off (men: <28 kg, women: <18 kg) as per the Asian Working Group for Sarcopenia (AWGS). Furthermore, since serum vitamin E is highly dependent upon circulating blood lipids [43], we adjusted total cholesterol in the regression model.

There are some limitations to our study. First, this study is cross-sectional in design; therefore, it is hard to distinguish a causal relationship between serum vitamin E level and HGS. Further prospective, controlled studies will be needed to assess the role of vitamin E in HGS. The second limitation is the lack of data on vitamin E consumption through daily diets or supplements. Vitamin E is mainly contained in nuts, seeds, and vegetable oils [44]. Even if the food is the same, it is difficult to quantify it through a food intake questionnaire because the vitamin E content varies depending on the recipe. In vitamin E supplements, because each supplement has a different dosage and it is impossible to determine the exact dosage for each individual, it is challenging to check the vitamin E consumption of all subjects. Due to the lack of information, we had to use the available data: crude supplement use. The third limitation is that there was no data on skeletal muscle mass or physical performance factors, such as 5-time chair stand test or 6-metre walk. According to the AWGS 2019 consensus update, the diagnostic criteria for sarcopenia include physical performance and appendicular skeletal muscle mass along with HGS [8]. Further studies will be needed to examinee directly the relationship between serum vitamin E and other sarcopenic index.

## 5. Conclusions

To summarize, we observed increased odds of low HGS at the lowest serum vitamin E quintile compared with the middle quintile in healthy Korean community-dwelling adults. Our study shows that lower level of serum vitamin E was associated with lower HGS, supporting the interest of vitamin E supplementation in low level subjects to prevent sarcopenia. Further well-controlled study trials are required to find an optimal concentration and supplementation of vitamin E for reducing the functional decline of muscle strength in the aging process.

## Figures and Tables

**Table 1:** Baseline characteristics of the study population according to serum vitamin E levels grouped by quintiles.

	Q1 (*n* = 429) <10.51 mg/L	Q2 (*n* = 431) 10.51–12.58 mg/L	Q3 (*n* = 429) 12.59–14.69 mg/L	Q4 (*n* = 434) 14.70–17.80 mg/L	Q5 (*n* = 431) ≥17.81 mg/L	*p*
Age (years)	64.5 ± 9.3	63.5 ± 9.4	63.2 ± 9.2	62.7 ± 8.4	62.0 ± 8.4	0.001
Sex, women	175 (40.8)	207 (48.0)	238 (55.5)	259 (59.7)	264 (61.3)	<0.001
Height, cm	162.2 ± 8.3	161.5 ± 8.7	160.6 ± 8.8	160.4 ± 8.8	160.1 ± 8.5	0.001
Weight, kg	63.7 ± 10.6	63.2 ± 10.8	62.2 ± 10.2	62.6 ± 10.6	62.2 ± 10.6	0.150
BMI, kg/m^2^	24.2 ± 3.4	24.1 ± 3.0	24.0 ± 3.0	24.2 ± 2.9	24.2 ± 3.1	0.923
Low handgrip strength ^a^	245 (57.1)	217 (50.3)	205 (47.8)	206 (47.5)	188 (43.6)	0.002
High household income(8.8 USD)	192 (44.8)	216 (50.1)	223 (52.0)	237 (54.6)	236 (54.8)	0.020
Educational status (≥high school)	223 (52.0)	228 (52.9)	231 (53.8)	236 (54.4)	240 (55.7)	0.847
Regular alcohol consumption	197 (45.9)	201 (46.6)	203 (47.3)	203 (46.8)	195 (45.2)	0.978
Current smoking	85 (19.8)	54 (12.5)	67 (15.6)	63 (14.5)	59 (13.7)	0.034
Resistance exercise						0.908
None	322 (75.1)	331 (76.8)	338 (78.8)	341 (78.6)	334 (77.5)	
Intermittent	51 (11.9)	51 (11.8)	40 (9.3)	47 (10.8)	47 (10.9)	
Regular	56 (13.1)	49 (11.4)	51 (11.9)	46 (10.6)	50 (11.6)	
Comorbidities						
Hypertension	215 (50.1)	207 (48.0)	197 (45.9)	197 (45.4)	199 (46.2)	0.625
Diabetes	111 (25.9)	79 (18.3)	78 (18.2)	72 (16.6)	82 (19.0)	0.006
Anemia	53 (12.4)	30 (7.0)	31 (7.2)	22 (5.1)	17 (3.9)	<0.001
Nutrition						
Total calorie intake, kcal/day	1909.9 ± 796.6	1971.0 ± 782.7	1908.5 ± 813.5	1834.1 ± 812.4	1861.6 ± 812.4	0.119
Total protein intake, g/day	63.9 ± 32.9	67.7 ± 32.8	65.2 ± 32.9	63.7 ± 32.9	65.8 ± 34.9	0.410
Use of dietary supplements	197 (45.9)	210 (48.7)	239 (55.7)	259 (59.7)	292 (67.7)	<0.001
Laboratory						
Fasting plasma glucose, mg/dL	106.4 ± 22.4	104.4 ± 23.2	105.7 ± 32.2	105.4 ± 24.2	107.6 ± 30.5	0.505
Total cholesterol, mg/dL	164.4 ± 33.5	183.9 ± 30.4	196.8 ± 34.1	207.4 ± 34.3	219.6 ± 42.2	<0.001
hsCRP, g/dL	0.60 [0.40, 1.19]	0.53 [0.36, 1.08]	0.60 [0.39, 1.15]	0.60 [0.40, 1.07]	0.68 [0.43, 1.23]	0.006

Results are expressed as number of participants (column percentage) for categorical variables and as average ± standard deviation or as median [interquartile range] for continuous variables. ^a^ Lower HGS than the sex-specific median values, <38.3 kg for men and <22.8 kg for women, respectively. Abbreviations: BMI, body mass index; hsCRP, high sensitivity C-reactive protein.

**Table 2:** Logistic regression models for low hand grip strength by serum vitamin E levels grouped by quintiles.

	Q1		Q2		Q3	Q4		Q5	
Regression Models	OR (95% CI)	*p*	OR (95% CI)	*p*	OR (95% CI)	OR (95% CI)	*p*	OR (95% CI)	*p*
Model 1	1.39 (1.03–1.87)	0.031	1.10 (0.82–1.48)	0.523	1.00 (reference)	1.39 (1.03–1.87)	0.799	1.10 (0.82–1.48)	0.675
Model 2	1.38 (1.02–1.86)	0.037	1.10 (0.82–1.48)	0.530	1.00 (reference)	1.38 (1.02–1.86)	0.769	1.10 (0.82–1.48)	0.704
Model 3	1.40 (1.02–1.91)	0.036	1.12 (0.83–1.51)	0.456	1.00 (reference)	1.40 (1.02–1.91)	0.778	1.12 (0.83–1.51)	0.544
Model 4	1.38 (1.01–1.89)	0.045	1.12 (0.83–1.52)	0.453	1.00 (reference)	1.38 (1.01–1.89)	0.834	1.12 (0.83–1.52)	0.560

Model 1 adjusted for age, sex, and BMI. Model 2 further adjusted for household income, alcohol consumption, current smoking, and frequency of resistance exercises. Model 3 further adjusted for hypertension, diabetes, serum total cholesterol, and log transformed hsCRP. Model 4 further adjusted for total calorie intake and the use of dietary supplements. Abbreviations: OR, odds ratio; CI: confidence interval; BMI, body mass index; hsCRP, high sensitivity C-reactive protein.

## Data Availability

Data are available from the Korea National Health and Nutrition Examination Survey (KNHANES VII), conducted by the Korea Centers for Disease Control and Prevention (KCDCP), and are freely available from KCDCP (https://knhanes.cdc.go.kr, accessed on 10 May 2021).
